# The Controversial C5a Receptor C5aR2: Its Role in Health and Disease

**DOI:** 10.1155/2017/8193932

**Published:** 2017-06-15

**Authors:** Ting Zhang, Malgorzata A. Garstka, Ke Li

**Affiliations:** Core Research Laboratory, The Second Affiliated Hospital, Xi'an Jiaotong University, Xi'an, Shaanxi, China

## Abstract

After the discovery of the C5a receptor C5aR1, C5aR2 is the second receptor found to bind C5a and its des-arginine form. As a heptahelical G protein-coupled receptor but devoid of the intracellular G*α* signal, C5aR2 is special and confusing. Ramifications and controversies about C5aR2 are under debate since its identification, from putative ligands and cellular localization to intracellular signals and pathological roles in inflammation and immunity. The ruleless and even conflicting pro- or anti-inflammatory role of C5aR2 in animal models of diverse diseases makes one bewildered. This review summarizes reports on C5aR2, tries to clear up available evidence on these four controversial aspects, and delineates C5aR2 function(s). It also summarizes available toolboxes for C5aR2 study.

## 1. Introduction

Being one of the major constituents of the innate immune system, complement plays an important role in protecting the body from pathogens, trauma, or the altered host milieu [1]. This phylogenetically ancestral system is comprised of sophisticated networks finely tuned in concert upon stimulation. The complement soluble zymogens, regulatory factors, and receptors function in an orchestrated way in response to harmful events. The three complement pathways converge on the proteolysis of C3. Complement fragments 3a and 5a (C3a and C5a), also termed anaphylatoxins, are released through the subsequent caspase cascades [2]. Of which, C5a, the most potent anaphylatoxin, mediates inflammatory immune responses such as chemotaxis, leukocyte degranulation, vascular permeability, cytokine and chemokine production, and functions beyond innate immunity in organ development, tissue regeneration, hematopoiesis, and others [3]. C5a is short-lived in circulation—its terminal arginine residue is rapidly cleaved by carboxypeptidases to generate relatively plasma-stable form called C5a des Arg. C5a and its des Arg form share partially coincident spectrum of biological activities [4]. When not properly controlled, they can aggravate local and systematic pathological processes, such as rheumatic arthritis, ischemia-reperfusion (I/R) injury, atherosclerosis, sepsis, and cancer [5–9].

Most of the C5a effects result from binding to the canonical complement 5a receptor 1, C5aR1. However, there is a second C5a receptor—C5aR2—that is thought to regulate the C5a-C5aR1 effects. C5aR2 is an enigmatic receptor; although discovered in 2000, so far, there is no unified theory about its biological and pathophysiological roles. Here, we review, quite often, contradictory evidence about possible function(s) of C5aR2 and attempt to answer whether it is a decoy receptor with anti-inflammatory properties and/or signaling receptor with pro- or anti-inflammatory properties. Moreover, we list mouse models, antibodies, agonists, and antagonists used to study C5aR2 in health and disease.

## 2. The Structure of C5aR2

In 2000 and 2001, the human *C5aR2* gene (also named *GPR77* or *C5L2*, *C5a-like receptor 2*) was cloned by two independent research groups [10, 11]. However, it was not until the works of Cain and Monk and Okinaga et al. that the receptor was characterized in terms of its ligand binding [12, 13]. It localizes on human chromosome 19q13.33-13.34, neighboring its paralog C5aR1. It encodes a protein of 337 amino acids in length that belongs to a subfamily of seven-transmembrane G protein-coupled receptors (GPCRs). The orthologs of C5aR2 in mouse and rat (mC5aR2 and rC5aR2, resp.) were subsequently cloned, and they share 61.3% and 56% sequence similarity to the human C5aR2 (hC5aR2), respectively, which is similar to the interspecies homology of C5aR1 [13–15].

**Figure 1:**
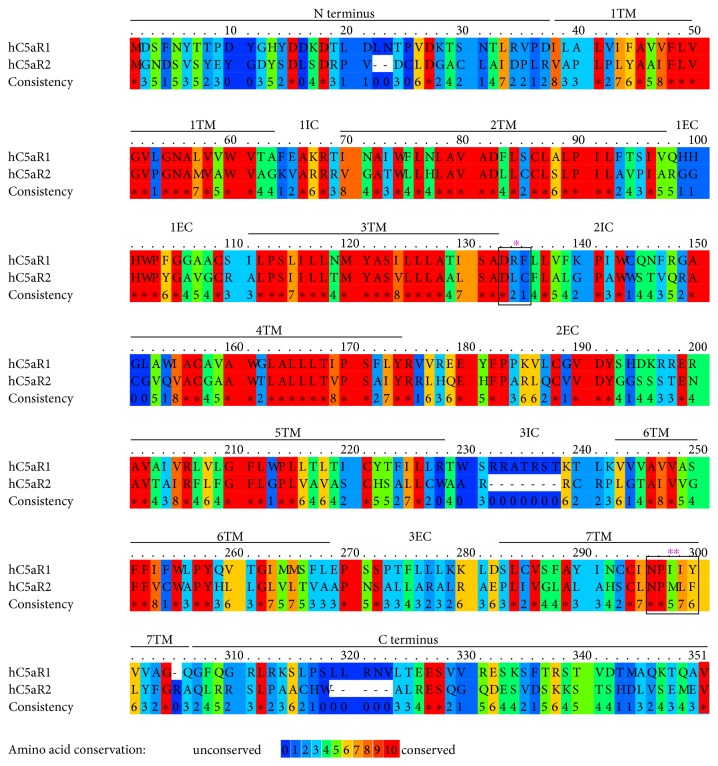
Amino acid sequence alignment of human C5a receptors. C5aR1 (top) and C5aR2 (bottom) were aligned using Praline (developed in the Centre for Integrative Bioinformatics Vrije Universteit Amsterdam). Transmembrane (TM) domains as well as intracellular (IC) and extracellular (EC) loops are indicated. Amino acid conservation is in the numeric representation. Regions important for G protein coupling in GPCRs are annotated as ∗ (DRY region) and ∗∗ (NPXXY region). Numbers at the right indicate the residue number.

The hC5aR2 shares 58% amino acid sequence identity with hC5aR1 and 55% with hC3aR in the transmembrane (TM) domains [10]. Similarly to C5aR1, C5aR2 possesses a single potential N-linked glycosylation site at Asn3 and is glycosylated, as suggested by the apparent molecular weight (45 kDa) of the receptor determined by SDS-PAGE and western blot relative to the predicted one (37 kDa) [13]. Possible N-linked glycosylation of C5aR2 may be important for its expression, ligand binding, and/or function. At the N-terminus, C5aR2 (similar to C5aR1) contains sulfated Tyr residues flanked by acidic amino acids that contribute to the formation of docking site of C5a. Moreover, both receptors are similar in charged and hydrophobic residues in their extracellular and transmembrane domains, suggesting an analogous ligand binding mode [16, 17]. C5aR1 and C5aR2 differ in three important regions that negatively affect the function of C5aR2: (i) change in the DRY motif (Asp-Arg-Tyr, highly conserved in many GPCRs) located after the third TM—the Arg residue essential for G*α* protein coupling is replaced by a Leu residue in C5aR2, (ii) shorter third intracellular loop lacking Ser/Thr residues that is the G protein recognition site in C5aR1, and (iii) change in NPXXY (Asn-Pro-X-X-Tyr) sequence in the seventh TM that acts as an internalization and signal transduction sequence in GPCRs (Figure 1) [13, 14, 18, 19].

## 3. Controversial Issue One: The Ligands of C5aR2—Is C5aR2 a Promiscuous Complement Fragment Receptor?

Amino acid alignment of C5aR2 and C5aR1 shows similar ligand-binding domain in N-terminus and ligand activation domain in the extracellular region [12, 13]. The ligand-binding assays performed by different research groups unequivocally proved that C5aR2 is a high-affinity receptor for both C5a and C5a des Arg ligands, thus comes its name—C5aR2—a second receptor (besides C5aR1) for C5a and C5a des Arg.

The hC5aR2 binds equally well to C5a and to des Arg form, whereas rodent C5aR2 orthologs prefer C5a des Arg, with much lower affinities for C5a [20]. According to some studies, both hC5aR2 and hC5aR1 bound C5a with a similar affinity; however, hC5aR2 had a 10- to 50-fold higher affinity than hC5aR1 for the C5a des Arg [12, 13]. Other studies reported that hC5aR1 might have similar affinities as hC5aR2 for both C5a and C5a des Arg [4, 21–23]. Scola and colleagues employed analogs, antibodies, and chimeric constructs and found that the pattern recognition sites of hC5aR2 and hC5aR1 were not the same despite conservation in several critical residues [20]. The hC5aR2, unlike hC5aR1, binds C5a and C5a des Arg using distinct mechanisms, with its N-terminus containing Tyr and acidic residues critical for binding of C5a des Arg but not of C5a.

However, the controversy arose whether C5aR2 could bind other ligands. When discovered as an orphan receptor and a member of the C5aR1 and C3aR family, C5aR2 was proposed to be the putative receptor for C4a and tested for binding of C4a, C4a des Arg, C3a, and C3a des Arg (also known as acylation-stimulating protein or ASP). Using RBL-2H3 (rat basophilic leukemia) cell line stably expressing *hC5aR2* gene and competitive binding of ^125^I-labeled ligand, Cain and Monk showed that C5a is a high-affinity ligand, while C3a and C4a are low-affinity ligands for C5aR2 [12]. Subsequently, using the same method, transiently transfected human embryonic kidney 293 (HEK293) cell line, and human skin fibroblasts, Kalant and colleagues demonstrated that C5aR2 could bind C3a and C4a, ASP, and C4a des Arg at a site distinct from the C5a-binding site [24]. As the long-sought functional receptor for ASP, C5aR2 together with ASP participated in lipid metabolism and glucose transport [25].

However, other studies provided contradicting evidence. Okinaga and colleagues used competitive ^125^I-labeled ligand-binding assay, but could not detect any interaction between C5aR2 and C3a or C4a in transiently transfected HEK293T (HEK293 cells transformed with large T antigen) or stably transfected murine pre-B lymphocytic cell line—L1.2 [13]. Kalant and colleagues attributed this discrepancy to the differences in experimental conditions employed: use of adherent versus suspension cells, 4°C or room temperature (RT) versus 37°C (a temperature at which both ligand-binding and ligand-receptor complex internalization occur), and limited competitor concentrations (0–10 *μ*M versus 0–300 nM) [25].

In the following years, pro and contra arguments contributed to a heated debate over this issue. The cons lodged their proofs. In 2006, Johswich and colleagues employed HEK293 and RBL cells either stably or transiently transfected with *C5aR2* gene and incubated at 4°C overnight according to the method of Kalant, pretreating filter plates with cationic protamine sulfate for the detection of ligand binding [26]. They found no specific binding of C3a and ASP to C5aR2, in accordance with Okinaga. C3a and ASP are basic peptides (pI 9.0) with high cationic charge, and could be sticky to plastic surfaces. To prevent it, tubes and tips used must be siliconized, or pretreated with cationic agents such as protamine sulfate or polylysine [26–28]. Most likely, the nonspecific binding of C3a and ASP to cells or plastic surfaces resulted in the false-positive results. In accordance with this, using transiently transfected Chinese hamster ovary (CHO) cell line and indirect immunofluorescence method, Scola and colleagues could not detect binding of C3a or ASP to hC5aR2 [20]. Neither could Croker and colleagues, when they utilized an Flp-In CHO cell line with *C5aR2* integrated into genome, and determined C3a and ASP binding by membrane and scintillation proximity assay [21].

The pros responded. Cui and colleagues addressed the inconsistencies between several previous studies [29]. They used cell lines overexpressing *C5aR2* gene (HEK and CHO cell lines transiently or stably, with either mock or *C5aR2* transfected), cells endogenously expressing *C5aR2* (3T3-L1, a cell line derived from mouse embryo fibroblasts), and no cells control under various experimental conditions (temperature: 4°C, RT, and 37°C; adherent versus suspension cells; different pretreatments and buffers: protamine sulfate, albumin, and others) and demonstrated specific binding of fluorescently or ^125^I-labeled ASP to both human and murine C5aR2 in a concentration-dependent manner. To exclude the potential C5a contamination in plasma ASP, recombinant ASP was tested in a competition assay, with a positive outcome. Commercial plasma ASP preparation involves denaturation, and it may be devoid of its activity and binding to the corresponding receptor [30]. Moreover, the concentration of recombinant ASP used by Cui shown to be bioactive was within physiological plasma ranges. In HEK cells stably transfected with *mC5aR2*, trafficking of the ASP-mC5aR2 complex was visualized using fluorescently conjugated ASP and anti-C5aR2 antibodies. Mouse C5aR2 underwent ASP-induced internalization and partially colocalized with ASP after 15 minutes and completely after 60 minutes—with slower kinetics than observed for C5a-induced internalization [31]. Employing antibodies recognizing different structural motifs, the first extracellular loop of C5aR2 was found to contribute to binding of ASP whose C-terminal was not essential for the interaction [32].

The experimental conditions to study C3a and ASP binding to C5aR2 must be established and unified. The multiple studies about the role of ASP and C5aR2 in lipogenesis supported the physiological relevance of these two. However, the question remains—whether ASP and C5aR2 interact directly.

## 4. The Expression of C5aR2

C5aR2 is expressed alongside C5aR1 with a broad expression pattern, although usually at a lower level [13, 33, 34]. The C5aR2 mRNA is detected in human, mouse, and rat cells and tissues of both myeloid and nonmyeloid origin, such as peripheral blood leukocytes, platelets, bone marrow, spleen, placenta, heart, lung, brain, anterior pituitary gland, ovary, liver, kidney, colon, thymus, small intestine, adipose tissue, and others [10, 11, 13, 14, 24, 33, 35, 36]. Human neutrophils are the most abundant source of C5aR2 [33]. It was reported that C5aR2 and C5aR1 mRNA levels were comparable in human monocyte-derived macrophages (HMDMs) [22]. Myeloid cell lines U937 and HL-60 (human promyelocytic leukemia cells) that upon stimulation can differentiate to macrophages, both expressing C5aRs only after Bt_2_cAMP treatment [26]. Tumor necrosis factor alpha (TNF*α*) did not affect *C5aR2* transcription in these cell lines.

Intriguingly, flow cytometry analysis of dendritic cells using polyclonal antiserum against N-terminal (23 amino acids) peptide of C5aR2 revealed hC5aR2 protein expression on immature dendritic cells, but not on mature dendritic cells [11]. Immunohistochemical analysis of human kidney biopsies showed expression of both receptors in renal tubuli, but their localizations were almost mutually exclusive; C5aR1 localized mainly in the Henle's loops, while C5aR2 in the distal convoluted tubuli 2 and the connecting tubuli [37]. This may suggest a functional role of hC5aR2 in dendritic cell maturation and in the kidney. Epithelial cervix adenocarcinoma (HeLa) cell line constitutively expresses abundant C5aR2, but not C5aR1, on its surface [26]. These two C5a receptors may share partially overlapping regulatory mechanisms.

Local complement activations in intestinal lumen have been reported [38]. And both C5aR1 and C5aR2 were detected exclusively on the apical surface of human intestinal epithelial cell lines [39]. It could be speculated that C5aRs on the luminal side of intestinal epithelium might participate in certain inflammatory pathological processes in response to C5a locally generated in the intestine.

However, the expression of C5aR2 in pathophysiological conditions needs further exploration. Neutrophils from patients with familial Mediterranean fever had lower C5aR2 mRNA expression [40]. The CD14^+^-circulating monocytes from tuberculosis patients expressed higher surface levels of C5aR2, compared to those from healthy donors [41]. But it was C5aR1, not C5aR2 who participated in the inhibition of Th1 polarization induced by highly pathogenic *Mycobacterium tuberculosis* M. strain. Both C5aR2 mRNA and protein have been detected in many cell types in human brain tissue, including various kinds of neurons, hippocampal pyramidal cells, glia, and astrocytes [10, 14, 42–44]. The upregulation of C5aR1 is proved to be detrimental in many neurodegenerative diseases, for example, Alzheimer's disease (AD), amyotrophic lateral sclerosis (ALS), and Huntington's disease (HD) [42, 45]. C5aR2 also accumulates in pathological areas of human AD and HD brains, either colocalizes with C5aR1 or not [43, 46]. In a rat model of ALS, C5aR2 was located mainly on motor neurons, and its levels first increased then declined with the disease progression, which was in line with motor neuron damage advance [42]. Yet in rat astrocytes, C5aR2 was upregulated by noradrenalin with a presumptive anti-inflammatory function [14]. In traumatic spinal cord injury mouse model, despite reduced intraparenchymal TNF*α* and interleukin-6 (IL-6) production, loss of C5aR2 worsened the outcome [47]. However, the expression and exact neuroprotective mechanisms of C5aR2 in spinal cord injury remain unclear. The role of C5aR2 in the central nervous system and its involvement in disease progression require further studies.

## 5. Controversial Issue Two: The Cellular Location of C5aR2

In resting human polymorphonuclear leukocytes (PMNs), peripheral blood monocytes (PBMs), and HMDMs, C5aR1 localizes predominantly on the cell surface, while C5aR2 mainly intracellularly [22, 33, 48, 49]. Similar distribution pattern is observed in murine neutrophils and macrophages [49, 50]. C5a, the chemokine (C-X-C motif) ligand 1 (CXCL1), and other treatments, which can alter cell activation status, could not induce C5aR2 expression on the surface of human neutrophils [33]. Upon C5a stimulation, C5aR1 was endocytosed and localized in the vesicles, and intracellular C5aR2 pool relocalized to C5aR1-positive vesicles. However, there are also reports showing expression of C5aR2 on myeloid cells. The surface C5aR2 on human neutrophils and monocytes shows large variations between individuals [18, 51]. The surface levels of C5aR2 on mouse and rat neutrophils may play a vital role in sepsis [7, 17, 52]. In mouse macrophage cell line RAW 264.7, C5aR2 was found predominantly on the cell surface [53]. In naïve and activated human CD4^+^ T cells, C5aR1 was exclusively intracellular and expressed at a low level, whereas C5aR2 was weakly expressed on the cell surface and abundantly in the cytoplasmic vesicles [54].

The discrepancy in C5aR2 location may be attributed to various cell lines/types chosen for investigation or the same cell line/type under different experimental conditions. The expression and localization of C5aR2 is species and cell specific, supported by the C5aRs distribution in different subpopulations of mast cells [55]. In human mast laboratory of allergic disease 2 (LAD2) cell line, C5aR2 localized at the cell surface, while C5aR1 intracellularly. C5a induced C5aR2 internalization, while IL-4 and stem cell factor, but not interferon gamma (IFNγ), increased its expression. CD34^+^ cell-derived primary mast cells expressed C5aRs intracellularly. In human mast cell line HMC-1, both receptors could be detected on the cell surface [51, 55]. The difference in cellular localization may lead to different ligand responses and thus receptor function.

## 6. Controversial Issue Three: Is C5aR2 a Decoy or Signaling Receptor?

Distinct from C5aR1 and C3aR, despite its ability to bind C5a and C5a des Arg, C5aR2 cannot elicit ligand-induced activation of heterotrimeric G proteins and downstream signals, for example, via MAPK pathway, nor mediate intracellular calcium fluxes and leukocyte degranulation [4, 13]. The alterations in certain important structural motifs mentioned above make C5aR2 obligatory uncoupled from G*α* protein. C5aR1 and C3aR undergo rapid ligand-dependent internalization. It seems that hC5aR2 is not internalized upon ligand binding, as determined by antibody detection of the remaining receptors on the cell surface after 5–10 minutes stimulation with ligand [12, 13]. Thus, initially, C5aR2 was considered as a nonsignaling “silent” receptor for C5a and C5a des Arg, acting as a “sink” for excess of C5a and especially C5a des Arg (due to its relatively high binding affinity) following complement activation, buffering C5a/C5a des Arg concentrations available for C5aR1 inflammatory responses.

Later, it was discovered that hC5aR2, when expressed in RBL cells, underwent constitutive internalization in a clathrin-dependent manner [18]. Human C5aR2 cycled between the endosomes and cell surface constitutively and independently on C5a/C5a des Arg. Thus, the surface levels of hC5aR2 remained constant. Meanwhile, C5a/C5a des Arg (particularly the latter) bound to hC5aR2 was targeted for lysosomal degradation in cells endogenously expressing *hC5aR2*, including Bt2cAMP differentiated HL-60 cells, HeLa cells, and human PMNs in a clathrin- and ATP-dependent manner. All this agreed with the paradigm for decoy and scavenger receptors. There are such known identified chemokine decoy receptors in GPCRs: D6 and DARC. C5aR2, as a decoy receptor for C5a/C5a des Arg, may compete with C5aR1 for ligand binding to dampen C5a inflammatory signaling, as observed in some pathological conditions [34, 56, 57].

However, these studies were mostly done in overexpression systems. Bamberg and colleagues questioned this hypothesis and used human PMNs naturally expressing both *C5aR1* and *C5aR2* and anti-C5aR2 mAbs: 4C8 and 1D9 [33]. C5aR2 was not found on the cell surface and its intracellular pool made no contribution to C5a sequestration from the extracellular space. Authors concluded that instead of being the proposed C5a-scavenging receptor, C5aR2 may function in modulating C5a signaling.

It is known that cytosolic *β*-arrestins not only mediate GPCRs desensitization and internalization but also couple to G protein-independent signaling pathways [58]. In fact, they serve as scaffolds for complex signaling networks [59]. There are two known isoforms of *β*-arrestins (*β*-arrestins 1 and 2). They may have distinct or shared roles for different GPCRs. Kalant and colleagues showed redistribution of GFP-tagged *β*-arrestin to C5aR2 after C5a/C5a des Arg stimulation [25]. The C5aRs (C5aR1 and C5aR2) colocalized with *β*-arrestin 1 in human PMNs after stimulation with C5a (100 nM) that suppressed C5aR1-mediated chemotaxis by inhibiting ERK1/2 activation, but had no effect on calcium fluxes. However, the colocalization of endogenous *β*-arrestin 1 with hC5aR2 could not be found in transfected RBL cell line stimulated with C5a [18]. In stably transfected HEK293 cells, C5a induced a *β*-arrestin 2-mediated C5aR2 internalization detected after 15 minutes stimulation, much slower than observed for C5aR1 [30]. Other studies showed *β*-arrestin-induced C5aR2 internalization in a C5a-independent manner [18, 33]. The association between hC5aR2 and *β*-arrestins may be constitutive [22, 33], and enhanced by C5a/C5a des Arg ligands binding [21, 22, 60], with binding of the des Arg form resulting in faster association of C5aR2 and *β*-arrestins [22]. *β*-Arrestin 2 preferentially bound to C5aR2 than to C5aR1. Nanomolar amounts of C5a/C5a des Arg were sufficient to recruit *β*-arrestin 2 to C5aR2, suggesting that this phenomenon might occur in vivo [60]. In HMDMs, interaction between C5aR2 and *β*-arrestin 2 inhibited C5a-induced ERK1/2 activation and lipopolysaccharide- (LPS-) induced IL-6 production [21].

C5aR1 may also be indispensable for C5aR2 signaling through *β*-arrestins. It is widely established that some GPCRs subtypes can form oligomers with not yet fully defined roles [61, 62]. C5aR1 has already been demonstrated to form homomers or heteromers with CCR5 [63, 64]. In vitro, high concentrations of C5a (100 nM–500 nM), but not of C5a des Arg, induced specific C5aR1-C5aR2 heteromer formation, modulated by C5aR2-*β*-arrestin 2 recruitment [21]. This, by downregulating ERK1/2 signaling, might contribute to the elevated expression of anti-inflammatory factor—IL-10 and granulocyte colony-stimulating factor (G-CSF) in HMDMs upon costimulation with LPS and 100–200 nM C5a [22, 23].

The proinflammation properties of C5aR2 in certain disease models further complicate the matter [7, 65, 66]. In bone marrow-derived mouse macrophages (BMDMs), neutrophils, and transfected HEK293T cells, C5aR2 interacted with C5aR1 and was required for C5a-induced C5aR1 endocytosis and downstream PI3K/ERK signaling in a clathrin adaptor AP2-depedent manner [67]. There is a motif—RXR (Arg-X-Arg) inside the first intracellular loop of human and murine C5aR2—that may interact with AP2 and recruit it to the C5aR1-C5aR2-*β*-arrestin 1 complex. C5a and C3a signaling pathways (including cytokine production, MAPK and PKB/Akt activation upon stimulation with C5a, actin polymerization, and ERK1/2 and Akt activation upon stimulation with C3a) were impaired in neutrophils and macrophages from C5aR2 KO mice [68]. Downregulation of surface C5aR2 rather than C5aR1 on human neutrophils soon after liver resection paralleled with decreased chemotaxis and _L_-selectin, Mac-1 expression of human PMNs [69]. Well, the author did not mention the alteration in intracellular C5aR2. In sepsis, C5aR2, not C5aR1, was responsible for the release of HMGB1, which was supported by an observation made by Croker and colleagues that high mobility group bow-1 (HMGB1) production from macrophages was independent on C5aR1-C5aR2 heteromer [22]. This suggests that unknown C5aR2 signaling pathways are still waiting to be discovered. Recently, Pundir and colleagues demonstrated that surface C5aR2 on human mast cell line LAD2 functioned in cell adhesion, migration, and proinflammatory mediators production in response to C5a and C5a des Arg (although weakly), which involved ERK, PI3K, and *β*-arrestin 2 pathways [55].

Transfection systems may not be an ideal tool to investigate GPCR signaling pathways due to deficiency of endogenous C5aR1 and possibly certain endogenous responsive machineries [70]. C5aR2 may weakly signal through Ca^2+^-irrelevant G proteins in some cell types endogenously expressing *C5aR2*. Likewise, besides their anti-inflammatory effect on C5aRs heteromer, *β*-arrestins can also recruit other components of signaling cascades, for example, c-Src and elicit GPCR-independent ERK and PI3K/Akt signals, which may cause inflammation under certain circumstances [59].

## 7. Controversial Issue Four: Is C5aR2 a Pro- or Anti-Inflammatory Receptor?

The existing publications have shown C5aR2 to have pro- and/or anti-inflammatory activities. And both functions are not mutually exclusive.

Proinflammatory properties of C5aR2 have been shown in experimental allergic asthma [65, 68], dextran sulfate sodium- (DSS-) induced acute colitis [67], thioglycollate-induced peritonitis and air-pouch inflammation [68], acute lung injury (AKI) [71], acute pyelonephritis [72], renal I/R injury [73], and sepsis [7]. In the mouse models of these diseases, depletion of C5aR2 alleviates the pathological symptoms, ameliorates acute inflammatory responses, and protects mice from severe tissue damage. In most cases, C5aR2 functions in parallel with C5aR1, indicating cooperation between C5aR2 and C5aR1 for full development of certain pathological injuries. In models of AKI induced by LPS, immune complexes, or C5a, the detrimental effects of C5aRs were through extracellular histones release in the lung [71]. In the mouse model of bilateral renal I/R, C5aR2 deficiency provided both functional and structural protection against renal I/R injury, better than C5aR1 deficiency [73]. Both renal- and leukocyte-expressing C5aR2 contributed to the detrimental effects on the kidney. Because of distinct expression pattern of C5aRs in renal tubuli [37], they might function independently, as indicated by differential gene expression profiles of inflammatory mediators. But whether the distribution of C5aRs in mouse kidney is similar to that in humans needs further confirmation. Meanwhile, C5aR2 did not affect leukocyte infiltration but could enhance neutrophil activation during renal I/R injury.

On the contrary, in allergic contact dermatitis [56] and LPS- [74] and immune complex- [34] induced lung injury, C5aR2 deficiency exacerbates inflammation and tissue injury in mice, providing evidence for the anti-inflammatory role of C5aR2. In oxazolone-induced contact dermatitis, C5aR2 knockout (KO) mice exhibited anabatic allergic sensitivity, increased neutrophil infiltration, and inflammatory mediators production (e.g., CXCL1/2, IFNγ, and IL-17A; both in vivo and ex vivo in lymph node cells) compared to those of WT mice, which was completely reversed following anti-C5aR1 mAb administration. In pulmonary immune complex-induced injury, C5aR2 deficiency resulted in significantly enhanced inflammatory indices, mainly in pulmonary inflammatory cell infiltration, lung tissue injury, and increased TNF*α* and IL-6 production in lung homogenates, together with increased in vitro chemotaxis of bone marrow cells towards C5a. The cross talk between nucleotide-binding oligomerization domain-like receptor with pyrin domain- (NLRP-) 3 inflammasome and intracellular C5 activation has been found, using C5aRs agonists and antagonists and T cells from patients with constitutively NLRP3 activation or mouse models of infection and autoimmunity [54]. In human CD4^+^ T cells, the C5-NLRP3 axis mediated IL-1*β* maturation, then IFNγ production and Th1 differentiation in an autocrine fashion, which was enhanced by intracellular C5aR1 but dampened by surface C5aR2. In vitro, blocking C5aR2 with antibody increased IL-8 level in C5a-treated intestinal epithelial cell lines, antagonizing the C5aR1 signal [39]. These are in support of the negative regulatory role of C5aR2 to suppress C5a-C5aR1 interaction. Meanwhile, C5aR2 activity is not confined to innate immunity, but serves as a critical constituent in adaptive immune regulation. Especially, in a mouse model of *Staphylococcus aureus* (*S. aureus*) bloodstream infection, C5aR1 and C5aR2 were both required in host defense, where they synergistically confined the inflammation in the kidney, while only C5aR1 was involved in bacteria clearance [50].

In fact, the dual functions of C5aR2 may occur simultaneously. More and more research supports this notion.

In two models of OVA- and house dust mite- (HDM-) induced allergic asthma, C5aR2 deficiency was protective, with reduced airway hyperresponsiveness and Th2 cytokine production. Different antigens led to slight distinct responses in pulmonary inflammation. In HDM-induced asthma, C5aR2 KO increased airway neutrophil infiltration as compared with that of WT controls and caused elevated IL-17A production. Together with in vitro studies of HDM-pulsed bone marrow-derived DC cells (mDCs), Zhang and colleagues suggested that C5aR2 might play complex dual roles in experimental allergic asthma—suppressing Th1 and Th17 differentiation through mDCs/T cells while inducing Th2 differentiation through other pulmonary cells [65].

In models of cecal ligation and puncture- (CLP-) induced polymicrobial sepsis, using antibodies blocking C5aRs or KO mice, both C5aRs played adverse roles [7]. In midgrade CLP, absence or blockade of either C5aR1 or C5aR2 was beneficial with improved survival outcomes and reduced proinflammatory mediators (e.g., IL-1*β*, MIP-1*α*, and MIP-2) in plasma, except elevated IL-6 in C5aR2 KO mice—consistent with another report of CLP-septic mouse model [17]. In cardiomyocyts (CMs) from CLP-septic C5aR1 and C5aR2 KO mice, both receptors contributed to the cytokine storm that have been linked to cardiosuppression in sepsis, demonstrated by reduced levels of IL-6, TNF*α*, IL-1*β*, IL-10, and others [75]. C5a signals via C5aRs also contributed to prolonged elevation of intracellular Ca^2+^ concentrations [76], activation of the NLRP3 inflammasome and induction of reactive oxygen species (ROS) [77] in CMs from CLP-septic mice. They caused reduced cardiac output as well as impaired cardiac function. Extracellular histones contributed to the cardiomyocyte dysfunction [78]. The appearance of extracellular histones in plasma in CLP-induced sepsis required both C5aRs, and was dependent on PMNs and NLRP3. Also, G-CSF release was C5aRs dependent during CLP sepsis in mice as well as peritoneal macrophages stimulated with high concentration of C5a (100 nM) and LPS in an Akt- and MEK1/2-dependent manner [79]. In high-grade CLP, only interception of both C5a-C5aRs signals was protective, but dual receptor blockade was invalid after the onset of sepsis, indicating that C5aR1 and C5aR2 cooperatively engaged during severe sepsis progression, especially at the early stage along with the immediate excessive production of C5a [80]. Dual C5aRs blockade could also abolish C5a-induced apoptosis of adrenomedullary cells in CLP-septic rats [81]. Meanwhile, release of HMGB1 by mouse phagocytes as well as human PBMCs and PMNs, both in vivo and in vitro, required C5a-C5aR2 signaling and downstream MAPK and Akt pathways [7]. Yet, it could not be established whether the activation of MAPKs was related to C5aR2 directly or indirectly as a result of cross talk between C5aRs and toll like receptors (TLRs). However, research on blood neutrophils in experimental and clinical sepsis showed that the downregulation of C5aR2 levels in PMNs demonstrated by western blot and flow cytometry was associated with a poor prognosis, indicating the protective role of C5aR2 in counterbalancing C5aR1 [57, 82]. Surface C5aR2 on neutrophils and peritoneal macrophages maintained by sphingosine kinase 1-sphingosine-1-phosphate (Sphk1-S1P) interaction dampened inflammation in endotoxin-induced sepsis in mice, in part through ERK1/2 [52]. In CLP-septic rats, Gao and colleagues found that C5aR2 increased in the lung, liver, and heart [17]. The cellular location of C5aR2 may determine its functions. And whether the regulation and function of C5aR2 in different organs in sepsis are similar to those in myeloid cells remains to be defined. C5aR2-KO mice were more susceptible to LPS-induced septic shock than WT mice, showing higher lethality and elevated serum IL-1*β* [68]. Stimuli with apiece properties may induce similar global inflammatory reactions, but distinct profiles of response mechanisms, thus influencing C5aR2 function.

There is cross talk between complement and TLRs [83]. Many studies provided evidence that C5aR1 synergized with TLRs in proinflammatory and immunoregulatory activities [84, 85]. PBMCs and whole blood cells preactivated with TLRs ligands (e.g., LPS) were hypersensitive to C5a and produced more proinflammatory cytokines such as IL-6 and IL-8 but less HMGB1 [86]. The underlying mechanism is proved to be TLRs' negative modulation of C5aR2, partially through reduction of C5aR2 expression, as seen in synergistic interaction between C5a and nucleotide-binding oligomerization domain- (NOD-) like receptor 2 [53]. The C5aR2 can either act as a negative modulator of TLRs-C5aR1-induced inflammation or can induce the release of proinflammatory HMGB1 [7, 86, 87]. Therefore, the net effect of C5aR2 function determines its role in different pathological conditions.

The conflicting observations about C5aR2 in disease models complicate the matter. In C57BL/6 mouse models of immune complex- and LPS-induced AKI, both pro- and anti-inflammatory properties of C5aR2 were found [34, 71, 74]. This inconsistency may result from the differences in the genetic background of C5aR2 KO mouse strains and/or immunogenic variations. In a mouse model of antineutrophil cytoplasmic antibody (ANCA) necrotizing and crescentic glomerulonephritis (NCGN) induced by anti-mouse myeloperoxidase (MPO), C5aR1 engagement was harmful, while C5aR2 had the protective and anti-inflammatory effect [88]. Hao and colleagues demonstrated in vitro that C5aR2 was proinflammatory in C5a-primed human neutrophils upon ANCA activation [89]. Blocking C5aR2 with specific antibody resulted in significant decrease in MPO concentration and membrane proteinase 3 expression in C5a-treated neutrophils and in reduced respiratory burst and degranulation of C5a- and ANCA-IgG-primed neutrophils. It is worth mentioning that the models supporting C5aR2's anti-inflammatory properties in AKI [34] and ANCA NCGN [88] employed the same C5aR2 KO and corresponding WT C57BL/6 mouse strains.

Also in atherosclerosis, the role of C5aR2 is less clear, in contrast to the clear-cut pathogenic role of C5aR1 [90–94]. C5aR2 expression in the aortas, spleen, and liver of female ApoE KO mice on chow diet was significantly lower compared to age-matched WT counterparts [94]. Furthermore, there was an increase in C5aR2 levels in mice aortas with age and lesion progression, comparing 25 to 9 and 12 weeks, respectively. However, it remains unclear whether there is a relationship between reduced C5aR2 expression and increased inflammatory milieu in ApoE KO mice. Contrarily, high C5aR2 expression in human atherosclerotic plaques reflected the advanced stages of atherosclerosis and correlated with excessive proinflammatory cytokine (TNF*α* and IL-1*β*) expression in human plaques [87]. In vitro experiments on BMDMs and PBMCs from C5aR1 and C5aR2 KO mice corroborated the requirement of both C5aRs in optimal cytokine production and leukocyte arrest. C5aR2 colocalized with C5aR1 and oxidized low-density lipoprotein in deep lesions of atherosclerotic plaques. In plaques, cell-expressing C5aR2 were mostly macrophages, less often neutrophils and endothelial cells. C5aR2 was absent in CD3^+^ T cells and vascular smooth muscle cells. This proatherogenic role of C5aR2 and its collaboration with C5aR1 were confirmed in vivo mouse models of wire-induced endothelial injury and high-fat diet-induced atherosclerosis [66]. Here, C5aR2 levels were elevated in aortas of wire-injured mice or mice on high-fat diet (12 weeks), both of atherosclerosis-prone ApoE KO. The proinflammatory and proatherosclerotic effects of C5aR2 manifested in neointima formation, increased plaque size and instability, and more macrophages and CD3^+^ T cells recruitment to the plaques, as well as increased C5aR1^+^ cells numbers and cytokine levels (TNF*α* and VCAM-1) in the plaques. Liu and colleagues investigated glycol- and lipometabolism in mice with different backgrounds fed with slightly distinct diet formula (high fat and high sugar compared to high fat) and found the protective role of C5aR2 in the development of atherosclerosis [95]. C5aR2 may have pathogenic role in this chronic inflammatory process of vascular complication. But the influence of diet on its function should be further investigated.

## 8. Other Functions

The functions of complement are not restricted to inflammation and immunity. The multifunctional C5a may be involved in tissue repair and regeneration. However, the reports about the participation of C5aR2 are less numerous. Recently, C5aR2 has been reported to repress brain-derived neurotrophic factor secretion from pulp fibroblasts induced by Gram-positive bacteria-derived lipoteichoic acid [96]. This negative anti-C5aR1 regulation by C5aR2 in dentin-pulp regeneration correlated with the upregulation of C5aRs and intracellular colocalization of C5aR1 with C5aR2 in inflamed pulp fibroblasts. In mice after sublethal dose of irradiation, the regeneration of hematopoietic cells was disturbed in C5aR2 KO mice compared to WT counterparts [68]. In a mouse model of partial hepatectomy, C5aR2 deficiency led to increased hepatic steatosis and necrosis, elevated mortality, and impaired liver regeneration [97]. Similarly, reduction of C5aR2 on human monocytes correlated with postoperative impairment of liver function [69]. As to cell migration and homing, C5aR2 was not responsible for C3a- and ASP-mediated enhancement of hematopoietic progenitor cell chemotaxis towards CXCL12 [98].

## 9. C5aR2 and ASP

In all the cells (also immune cells), metabolism is required for various cellular processes, production of cellular building blocks, and waste removal [99]. More and more studies investigate the interaction between the immune system (complement system as well as other immune system components) and metabolism—so-called immunometabolism [100, 101]. The involvement of C5aR2 in obesity and related pathologies has long been reported and summarized in detail in several reviews [2, 5, 102, 103].

The des Arg form of C3a‐ASP functions as a lipogenic hormone, stimulates triglyceride (TG) synthesis by raising diacylglycerol acyltransferase activity, and promotes glucose transport through increasing glucose transporter translocation to the plasma membrane in adipocytes and preadipocytes in an autocrine and/or paracrine fashion. C5aR2 is expressed also in fat tissue. The Cianflone group investigated C5aR2 interaction with ASP and found that following ASP stimulation, hC5aR2 in transfected HEK cells underwent strong phosphorylation after 15 minutes and internalization and colocalization with *β*-arrestin 2 after 60 minutes [25, 30]. The G*_β_*- and Gq-mediated activation of phospholipase C, PI3K, and downstream protein kinase C, Akt, and MAPK were involved in ASP-mediated signaling [104, 105]. Gain/loss-of-function studies showed the relationship between C5aR2 and ASP in cells overexpressing and endogenous expressing *C5aR2* [106]. Surface C5aR2 expression increased when preadipocytes 3T3-L1 differentiate to adipocytes [31]. Factors which can downregulate C5aR2 also decreased the responses of 3T3-L1 adipocytes to ASP [104, 107, 108]. Human liposarcoma cell line (SW872) with no surface expression of C5aR2 showed no ASP-induced signaling [109]. C5aR2 KO mice displayed reduced adipose tissue triglyceride (TG) synthesis and fatty acid reesterification, increased plasma glucose clearance on both chow and high fat diet, with distinct metabolic features concerning the content of dietary fat [106]. The metabolic profiles of increased food intake, delayed postprandial TG clearance and elevated energy expenditure were similar between C5aR2 KO and C3 (ASP precursor) KO mice. Concordant results were shown when blocking antibodies against ASP or C5aR2 were administrated for ten days [32]. This short-term investigation was designed to avoid potential compensatory alteration of body weight and hormone levels in mice inherently lacking C5aR2 or ASP [110]. Taking a closer look, the effects of C5aR2 deficiency on TG mass and relevant enzyme activities differed among tissues, with lipid redistribution towards skeletal muscle for oxidation [32, 106, 111]. The apparently accelerated energy expenditure and altered substrate partitioning in the context of C5aR2 disruption turned into aggravated metabolic dysfunction and inflammation under obese milieu. C5aR2 KO mice on a diabetogenic diet developed stronger insulin resistance than WT mice through substrate redistribution, aberrant fat deposition, as well as systemic inflammation [112]. Macrophage infiltration in adipose tissue and diverse cytokines in plasma were all elevated in C5aR2 KO mice on a fat-enriched diet, which coincided with the chronic low-grade inflammatory state in obesity-associated disorders [112, 113]. In type II diabetes mellitus (T2DM) patients, skeletal muscle C5aR2 protein levels were reduced compared to those in obese controls—the reciprocal interaction of C5aR2 expression and muscle function changes induced by an insulin resistant state [111]. C5aR2 gene single nucleotide polymorphisms were found to be associated with metabolic disorders in different populations, including hyperlipidemia, T2DM, and coronary heart disease [114–119]. The S323I (Ser to Ile) mutation in the Ser-Thr rich carboxyl terminal of C5aR2 was found to be associated with a familial combined hyperlipidemia, probably due to the loss of function by this mutant in mediating ASP-induced signaling [30, 114].

The cross talk between C5a-C5aR1/C5aR2 and ASP-C5aR2 may exist. In adipocytes and macrophages, C5aR2 and C5aR1 colocalized upon either ASP or C5a stimulation [31]. The colocalization was both enhanced in these two important cell types in fat depots when cultured in media collected from coculture of both cells, each cell type had its specific colocalization and signal responding profile against conditioned medium. C5a-C5aR1 and ASP-C5aR2 signals acted in concert to regulate immune and metabolic responses in adipose tissue [120]. The association of C5aR2/C5aR1 with metabolic processes was also found in obese women with age-matched controls as well as obese women pre- and postbariatric surgery [121, 122].

Human mast cell LAD2 with surface C5aR2 responses to ASP led to degranulation, adhesion, and proinflammatory mediator production in an ERK-dependent manner [55]. In short, C5aR2 may be involved in metabolic inflammation, so-called metaflammation.

## 10. Tools for C5aR2 Study

For C5aR2 function investigation, C5aR2 KO mice, antibodies recognizing C5aR2, and agonists and antagonists of C5aR2, as well as transfection and RNA interference systems were employed. Tables 1–6 list and summarize tools used by different research groups. Mouse models are shown in Tables 1–3, C5aR2 agonists and antagonists in Table 4, and antibodies in Tables 5-6.

Lots of peptidic or nonpeptidic agonists and antagonists for C5aR1 have been discovered, making them efficient tools for C5aR1 functional studies [130, 131]. However, the agonists and antagonists for C5aR2 are lacking. Till now, no specific antagonist for C5aR2 has been found or designed. Jun/fos-A8 and A8^△71–73^, C5a mutant peptides based on site-directed mutagenesis, are the antagonists that can block the binding of C5a and C5a des Arg to both human and mice C5aRs [51]. Jun/fos-A8 is also reported to compete for ASP binding to C5aR2 [29]. In a two-stage de novo protein design framework, two peptides derived from C-terminus of C5a were identified as agonists for C5aR2 [127]. These two ligands, named P32 and P59, despite their low affinity for hC5aR2, could strongly stimulate the association of hC5aR2 with *β*-arrestin 2 without activating hC5aR1 G protein signal, when used at micromolar concentrations [21, 127].

Recently, naturally existing pore-forming toxins from *S. aureus* isolates—γ-hemolysin CB (HIgCB) and one subunit of Panton-Valentine leukocidin- (PVL)- LukSPV—were found to target C5aRs with species specificity, which may provide insights into the binding characteristics of its ligands to C5aR2 [48, 128, 129].

Specific ligands modulating the activity of C5aR2 are valuable tools for receptor function evaluation [7, 50, 52, 54]. However, the mechanisms underlying C5aR2 agonism and antagonism remain less clear than those of C5aR1. More such agents need to be found, which will play a key role in exploring the potential signaling mechanisms of C5aR2 and could be used as therapeutic drugs targeting C5aR2.

Targeting C5aR2 by RNAi, shRNA, or CRISPR-Cas9 can be another effective tool to silence C5aR2 downstream signaling [14, 25, 55, 96]. There are available reagent kits to downregulate or knock out *C5aR2* gene expression.

## 11. Conclusion

The research of the past decade is gradually unraveling the veil over C5aR2—this Janus-faced receptor. Since its discovery, controversies lay in multifaceted aspects of C5aR2, from putative ligands, cellular localization, to intracellular signals and pathological roles in inflammation and immunity. This confusing and enigmatic receptor is unlike any ordinary and simple receptor that was initially expected to be. It is us who are fettered by preconceived notions.

In transfected cells, C5a/C5a des Arg are demonstrated to be the cognate ligands for C5aR2. The binding of ASP to C5aR2 is still in debate, albeit animal and clinical data supporting the involvement of both in obesity and in metabolism.

In cells naturally expressing *C5aR2*, its expression and localization are likely to be dynamic and cell specific, undergoing continuous changes and dependent on diverse milieus, which may determine the ambivalent functions of C5aR2. When localized on the cell surface, C5aR2 may serve as a nonsignaling default receptor or decoy receptor sequestering C5a/C5a des Arg from C5aR1. Under those circumstances, C5aR2 may assume an anti-C5aR1 character, negatively modulating the C5a-C5aR1 signals. There is still the possibility that surface C5aR2 may also serve as a functional receptor mediating ligand-induced signals through certain signaling pathways. While restricted predominantly to the cytoplasm, due to the spatial separation between intracellular C5aR2 and extracellular C5a/C5a des Arg, direct interaction is not possible. C5aR2 can still function through intracellular network(s) with C5aR1 and other proteins such as *β*-arrestins. Chen and colleagues suggested the involvement of C5aR2 in optimization of C5a-C5aR1 and C3a-C3aR signals, proposing the formation of heterodimers of C5aR2 with either C5aR1 or C3aR [68]. Dimerization of C5aR2 and C3aR is yet to be confirmed. Under physiological conditions, C5aR1 levels on blood neutrophils are comparable between WT and C5aR2-null mice and vice versa [7]. But on neutrophils from bone marrow of C5aR2 KO mice, the surface expression of C5aR1 is lower than on those from WT mice [68]. Surface C5aR1 expression on nonstimulated BMDMs from C5aR2 KO mice is lower compared with those from WT littermates, also observed for C5aR2/ApoE double KO [66]. C3aR expression levels in neutrophils were similar between C5aR2 KO and WT mice [68]. So, in both physiological and pathological conditions, the relationship between all three receptors' expression needs further investigation. On the basis of the available experimental data, Li and colleagues listed three possible mechanisms of C5aR2 function [49]. Two hypotheses describe the anti-inflammatory roles of C5aR2: first—acting as a decoy receptor—C5aR2 sequesters C5a and C5a des Arg from C5aR1 and second—C5aR2 blocks C5aR1 signaling via a *β*-arrestin pathway. The third hypothesis suggests the proinflammatory role of C5aR2, although the intracellular signals are not yet precisely defined. These three hypotheses may not be mutually exclusive if viewed from a dynamic perspective.

It has been shown for C3aR and other GPCRs that there is a difference between the surface- and intracellularly induced signaling, which may also apply to C5aR2 [132]. The dynamic ratio of surface to the intracellular expression of C5aR2 depending on the cell type and/or normal versus pathological conditions determines the way it may function. C5aR2 has been shown to heterodimerize with C5aR1, mediating either pro- or anti-inflammatory pathways. This may result from C5aR1 versus C5aR2 activation balance or depend on the concentrations of C5a. High local concentration of C5a induces heterodimerization of C5aRs facilitating anti-inflammatory cytokines production, which could be the protection mechanism from excessive inflammation [22, 23]. “Biased agonism” has been proposed in the light of the evidence that different ligands for the same receptor can induce different responses [133]. Natural agonists elicit different or partially overlapping C5aR1 signaling compared with that induced of C5a [134, 135]. C5a, C5a des Arg, or the still inconclusive ligand ASP, as well as cross talks with the diverse surface or intracellular signals, may have a similar effect on C5aR2. There is a C5aR2-dependent upregulation of HMGB1 in sepsis both in vivo and in vitro, while in the context of cerebral malaria induced by *Plasmodium falciparum* it is C5aR1, but not C5aR2, that causes the elevated plasma HMGB1 [124].

C5aR2, like a bilateral switch in the complement system, appears to propagate inflammation in one situation or attenuate the phlogistic state in another, seemingly elusive and haphazard. Furthermore, it may have functions beyond immunity—in metabolism and regeneration. Perhaps these pro- or anti-inflammatory functions exist simultaneously in one cell type or in different cells, tissues, and organs in a counterbalanced way and the role of C5aR2 is defined by the net effect.

However, it is still unclear about the downstream consequences and regulatory mechanisms of C5aR2 that seem to be cell, condition, or even species specific. Although some remain debatable, previous studies about C5aR2 have shed light on the directions for our future research. Obviously, we could not expect to answer all the questions about C5aR2 in one essay. Rome was not built in a day. We should go step by step, cut across this moor, gradually unravel the role of C5aR2 at the molecular level, then cellular, tissue, organ, and whole organism integrally, some of which we have already done. Moreover, substantial differences in the complement regulatory pathways exist between species [54]. It is crucial to verify the role of C5aR2 found in rodent models in human, which will give accurate implication for clinic intervention.

## Figures and Tables

**Table 1:** C5aR2 KO on BALB/c background.

Source	Disease model	Properties of C5aR2	Refs
J. Khöl's laboratories, University of Lübeck, Germany	OVA- and HDM-induced experimental allergic asthma	Dual functions: anti-inflammation on mDCs, proinflammation on pulmonary cells	[65]
	TLR induction in vitro and in vivo	Dual functions: anti-inflammation—C5aR2 negatively modulates TLRs-C5aR1 on PBMCs and whole blood cells; proinflammation—C5aR2 promotes HMGB1 expression	[86]
	Peritoneal membrane fibrosis	No function	[123]
TL31 KO from Amgen, South San Francisco	DSS-induced acute colitis	Proinflammation	[67]
Lexicon Genetics, the Woodlands, Texas	OVA-sensitized, methacholine-induced asthma	Proinflammation	[68]
Dr. Craig Gerard, Harvard Medical School—C57BL/6 mice backcrossed to the BALB/c background	OX-induced experimental allergic contact dermatitis	Anti-inflammation	[56]
	LPS-induced acute lung injury	Anti-inflammation	[74]

**Table 2:** C5aR2 KO on C57BL/6 background.

Source	Disease model	Properties of C5aR2	Refs
B. Lu, Harvard Medical School, USA	IC-induced acute lung injury	Anti-inflammation	[34]
	Anti-mMPO-induced ANCA NCGN	Anti-inflammation	[88]
	CLP-induced sepsis and in vitro assays on leukocytes	Proinflammation and indispensable for HMGB-1 release	[7]
	CLP-induced sepsis	On CMs—proinflammation and causes cardiac dysfunction	[75, 76, 78]
	Acute pyelonephritis	Proinflammation	[72]
	Renal I/R injury	Proinflammation	[73]
	Experimental cerebral malaria	No function^※^	[124]
	*S. aureus* bloodstream infection	Anti-inflammation	[125]
	AKI induced by LPS, IC, or C5a	Proinflammation	[71]
Genotyping and breeding in University of Michigan according to the method of Dr. Craig Gerard	CLP-induced sepsis	Indispensable for G-CSF release by macrophages	[79]
	CLP-induced sepsis	On CMs—proinflammation: activation of the cardiac NLRP3 inflammasome	[77]
Professor A. Klos, Hannover Medical School, Germany	Wire-induced endothelial denudation of the carotid artery, diet-induced atherosclerosis	Proinflammation	[66]
The Jackson Laboratory	In vitro atherosclerosis model	On PBMCs and BMDMs—proinflammation	[87]
Lexicon Genetics, the Woodlands, Texas	Thioglycollate-induced peritonitis and air-pouch inflammation	Proinflammation	[68]
	LPS-induced septic shock	Anti-inflammation	[68]

※ means that C5a-C5aR1 contributes to the cerebral [124] and placental [126] malaria pathogenesis in human and mouse, rather than to C5a-C5aR2 signal.

**Table 3:** C5aR2 KO with lipometabolism.

Source	Disease model	Properties of C5aR2	Refs
C5aR2 heterozygous mice from Regeneron Pharmaceutics Inc.	Physiological metabolism of C5aR2 KO mice	Stimulates TG synthesis and glucose transport in adipose tissue	[106]
	Diet induced obesity	Anti-inflammation	[95, 113]
	Partial hepatectomy	Promotes liver regeneration	[97]
	High fat diet-induced insulin resistance	Anti-inflammation, metabolic regulation	[112]

**Table 4:** C5aR2 agonists and antagonists.

Ligand	Specificity	Function	Refs
Jun/fos-A8 Jun/fos-A8^△71–73^ C5 mutant peptides	Human and murine C5aR1 and C5aR2	Antagonists—block binding of C5a and C5a des Arg to C5aRs	[51]
P32, P59—C terminal peptides of C5a	hC5aR2	Agonists—strongly stimulate the association of hC5aR2 with *β*-arrestin 2	[21, 127]
LukS-PV—protein from *S. aureus*	Human and rabbit C5aR1 Human, macaque, and rabbit C5aR2	Antagonist of C5a-induced activation of neutrophils	[48, 128]
HIgCB—protein complex from *S. aureus*	Human, macaque, rabbit, and cow C5aRs	Antagonist of C5a-induced activation of neutrophils	[128, 129]

**Table 5:** Antibodies against human C5aR2.

Antibody	Type (source)	Applications	Refs
hC5aR2 N1-23	Rabbit polyclonal (Hycult)	FACS, WB, IF, IHC on frozen or paraffin sections	[37, 46]
hC5aR2 N 1-50	Rabbit polyclonal (Imgenex)	WB, ICC, IF, IHC	[22, 23, 33]
hC5aR2 N 1-50	Rabbit polyclonal (Abcam)	WB, IHC on paraffin sections	[46]
hC5aR2 C 275-325	Rabbit polyclonal (Novus)	IHC on paraffin sections	[46]
hC5aR2 248-311	Rabbit polyclonal (Abcam)	WB	[46]
hC5aR2 clone 1D9M12	Mouse monoclonal (Biolegend)	FACS, WB, blocking	[46, 48, 86, 89]
hC5aR2 clone 4C8	Mouse Monoclonal (T. Woodruff)	FACS, blocking, IHC on paraffin sections	[33, 46]
hC5aR2 N3C1 internal	Rabbit polyclonal (Gene Tex)	IHC on frozen or paraffin sections	[87]
hC5aR2-L1 (extracellular loop one)	Rabbit polyclonal (K. Cianflone)	Blocking ASP-C5aR2 interaction	[32]

WB: western blot, FACS: flow cytometry, IF: immunofluorescence, IHC: immunohistochemistry, ICC; immunocytochemistry.

**Table 6:** Antibodies against rodent C5aR2.

Antibody	Type (source)	Applications	Refs
mC5aR2 N1-23	Rabbit polyclonal (K. Cianflone)	FACS, IF	[25]
mC5aR2 N1-38	Rabbit polyclonal (P. A. Ward)	FACS, blocking, WB, ELISA for both mouse and rat C5aR2	[7, 17, 57, 81]
rC5aR2 N1-29	Rabbit polyclonal (P. G. Noakes)	WB, IF, IHC on frozen sections	[42]
mC5aR2	Rabbit polyclonal (Hycult)	FACS	[50, 52]

WB: western blot, FACS: flow cytometry, IF: immunofluorescence, IHC: immunohistochemistry, ELISA: enzyme-linked immunosorbent assay.
