# Corrigendum to “The Controversial C5a Receptor C5aR2: Its Role in Health and Disease”

**DOI:** 10.1155/2017/6073961

**Published:** 2017-11-01

**Authors:** Ting Zhang, Malgorzata A. Garstka, Ke Li

**Affiliations:** Core Research Laboratory, The Second Affiliated Hospital, Xi'an Jiaotong University, Xi'an, Shaanxi, China

In the article titled “The Controversial C5a Receptor C5aR2: Its Role in Health and Disease” [1], there was an error in Table 4, where Jun/fos-A8^△71–73^ should be corrected to A8^△71–73^. The corrected table is as follows:

## Figures and Tables

**Table 4:** C5aR2 agonists and antagonists.

Ligand	Specificity	Function	Refs
Jun/fos-A8 A8^△71–73^ C5 mutant peptides	Human and murine C5aR1 and C5aR2	Antagonists—block binding of C5a and C5a des Arg to C5aRs	[51]
P32, P59—C terminal peptides of C5a	hC5aR2	Agonists—strongly stimulate the association of hC5aR2 with *β*-arrestin 2	[21, 127]
LukS-PV—protein from *S. aureus*	Human and rabbit C5aR1 Human, macaque, and rabbit C5aR2	Antagonist of C5a-induced activation of neutrophils	[48, 128]
HIgCB—protein complex from *S. aureus*	Human, macaque, rabbit, and cow C5aRs	Antagonist of C5a-induced activation of neutrophils	[128, 129]
